# Supplementary material for: A dataset for evaluating clinical research claims in large language models
Source: Sci Data. 2025 Jan 16;12:86. doi: 10.1038/s41597-025-04417-x (PMC11739414; doi:10.1038/s41597-025-04417-x)
Supplement: Supplementary file 1 — Supplementary Information [file 41597_2025_4417_MOESM1_ESM.pdf]

Supplementary Information

Algorithm S1 Claim-evidence labeling

```
// Set of clinical trials
CT = {ct_1, ct_2, ..., ct_m}

for each ct in CT:

   // Retrieve all claims associated with the clinical trial
   Claims = {C_1(ct), C_2(ct), ..., C_n(ct)}

   for each claim C_n(ct) in Claims:

      // Retrieve the p-value for the primary outcome measure
      p_value = get_p_value(ct, C_n(ct))

      // Step 1: Assign positive or negative label based on p-value
      if p_value < 0.05:
         L_1[C_n(ct)] = "positive"
      else:
         L_1[C_n(ct)] = "negative"

   // Retrieve all abstracts linked to the clinical trial
   E_ct = get_linked_abstracts(ct)

   // Separate abstracts that report results and those that provide background information
   result_abstracts = {e in E_ct | e reports results for ct}
   background_abstracts = {e in E_ct | e provides background information}

   // Step 2: Determine the nature of the link and assign labels accordingly
   if size(result_abstracts) == 1:
      // Exactly one abstract reports results for the trial
      e = the single abstract in result_abstracts

      // For each claim, assign L_2 based on L_1
      for each claim C_n(ct) in Claims:
         if L_1[C_n(ct)]  == "positive":
            L_2[(C_n(ct), e)] = "evidence"
         else:
            L_2[(C_n(ct), e)] = "inconclusive"

   if size(background_abstracts) >= 1:
      // Abstracts provide background information
      for each claim C_n(ct) in Claims:
         for each e in background_abstracts:
            L_2[(C_n(ct), e)] = "not enough information"
```

Table S1: Mapping arm types to intervention or comparator.

**Mapped Term**	**Arm Type**
Intervention	Experimental
Comparator	Active Comparator
	Placebo Comparator
	Sham Comparator
	No Intervention
	Other

Figure S1: Number of publications per year in CliniFact.

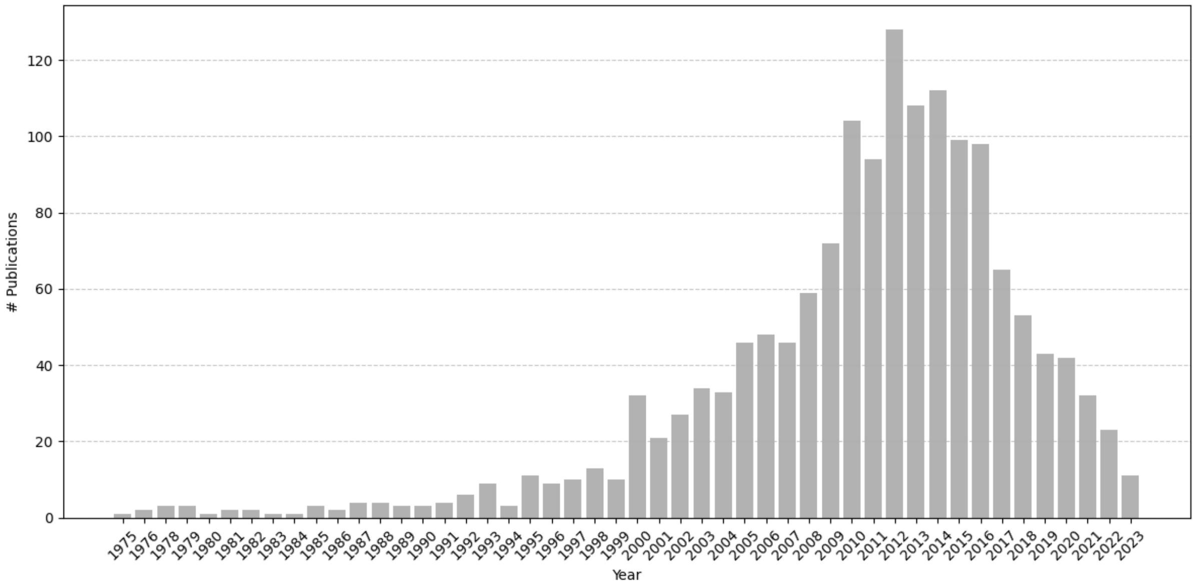

Table S2: Data Structure of CliniFact dataset.

**Variable**	**Column Heading**	**Description**
CliniFact Index	index	A unique identifier to each instance.
Clinical Trial Identifier	nctId	The unique CT identifier.
Claim	claim	The scientific claim derived from CT.
PubMed Identifier	PMID	The unique PubMed ID.
Article Title	article_title	The publication title.
Article Abstract	article_abstract	The publication abstract.
Hypothesis Label	label	Whether the abstract provides evidence for the

		claim, is inconclusive, or contains not enough information (NEI).
Outcome Measure Type	outcome_type	The type of the measured outcome (e.g., primary, secondary).
Outcome Measure Title	outcome_title	The title of the measured outcome.
Outcome Measure Description	outcome_description	A detailed explanation of the measured outcome.
Arm/Group Title	intervention_group_title	The name of the intervention group.
Arm/Group Title	comparator_group_title	The name of the comparator (control) group.
Arm Description	intervention_group_description	A description of the intervention.
Arm Description	comparator_group_description	A description of the comparator.
Intervention Type	intervention_group_intervention_label	The type of the intervention (e.g., drug, device)
Comparator Type	comparator_group_intervention_label	The type of the comparator (e.g., placebo)
Arm Type	intervention_group_arm_group_type	The type of the intervention arm (e.g., experimental).
Arm Type	comparator_group_arm_group_type	The type of the comparator arm (e.g., placebo comparator, active comparator).
Estimated Value	pValues	The p-value.
Method	statisticalMethods	The statistical method.

Type of Statistical Test	nonInferiorityTypes	The statistical test type (e.g., non-inferiority, equivalence, superiority).
Article Publication Date	Publication Date	The date of publication.

Figure S2: Flowchart of the truncation algorithm.

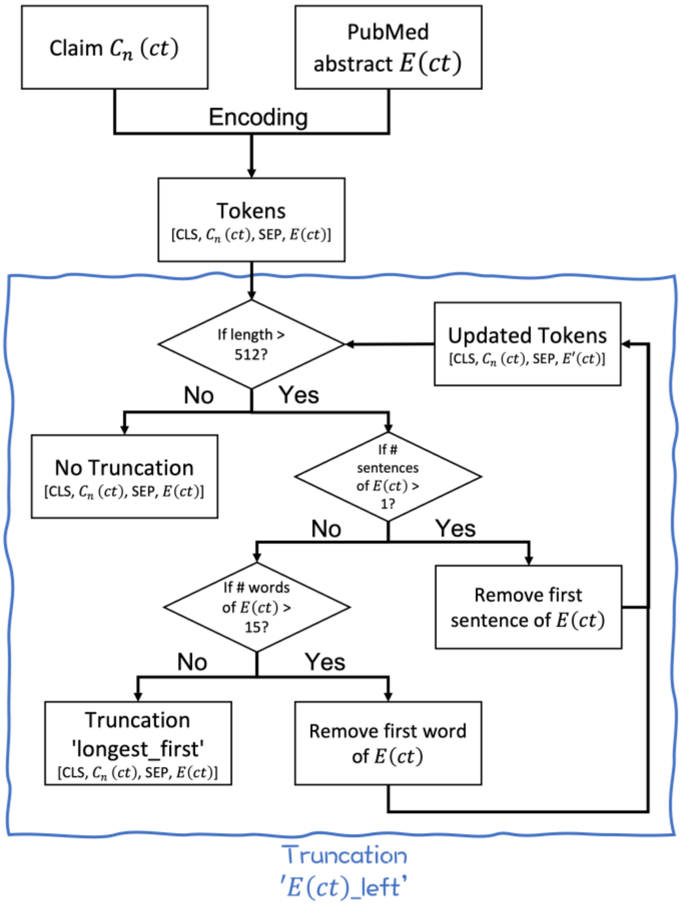

We first truncate from the left side of the abstract—removing entire sentences and then, if necessary, up to 15 words from the start of the last sentence—because the later section of abstracts typically contains the evidence for claim verification. If we still exceed 512 tokens after these steps, we revert to the default truncation method.
